# Internet use, eHealth literacy and attitudes toward computer/internet among people with schizophrenia spectrum disorders: a cross-sectional study in two distant European regions

**DOI:** 10.1186/s12911-017-0531-4

**Published:** 2017-09-20

**Authors:** Christina Athanasopoulou, Maritta Välimäki, Katerina Koutra, Eliisa Löttyniemi, Antonios Bertsias, Maria Basta, Alexandros N. Vgontzas, Christos Lionis

**Affiliations:** 1 Department of Nursing Science, Faculty of Medicine, University of Turku, Turku, Finland; 2 Turku University Hospital, Turku, Finland; 3 Hong Kong Polytechnic University, Kowloon, Hong Kong; 4 Department of Psychiatry & Behavioral Sciences, Faculty of Medicine, University of Crete, Heraklion, Greece; 5 Biostatistics Unit, Faculty of Medicine, University of Turku, Turku, Finland; 6 Clinic of Social and Family Medicine, Faculty of Medicine, University of Crete, Heraklion, Greece

**Keywords:** Schizophrenia, Mental illness, Internet, Computers, Technology, eHealth literacy, Attitudes, Interest, Efficacy, Use

## Abstract

**Background:**

Individuals with schizophrenia spectrum disorders use the Internet for general and health-related purposes. Their ability to find, understand, and apply the health information they acquire online in order to make appropriate health decisions – known as eHealth literacy – has never been investigated. The European agenda strives to limit health inequalities and enhance mental health literacy. Nevertheless, each European member state varies in levels of Internet use and online health information-seeking. This study aimed to examine computer/Internet use for general and health-related purposes, eHealth literacy, and attitudes toward computer/Internet among adults with schizophrenia spectrum disorders from two distant European regions.

**Methods:**

Data were collected from mental health services of psychiatric clinics in Finland (FI) and Greece (GR). A total of 229 patients (FI = 128, GR = 101) participated in the questionnaire survey. The data analysis included evaluation of frequencies and group comparisons with multiple linear and logistic regression models.

**Results:**

The majority of Finnish participants were current Internet users (FI = 111, 87%, vs. GR = 33, 33%, *P* < .0001), while the majority of Greek participants had never used computers/Internet, mostly due to their perception that they do not need it. In both countries, more than half of Internet users used the Internet for health-related purposes (FI = 61, 55%, vs. GR = 20, 61%). The eHealth literacy of Internet users (previous and current Internet users) was found significantly higher in the Finnish group (FI: Mean = 27.05, SD 5.36; GR: Mean = 23.15, SD = 7.23, *P* < .0001) upon comparison with their Greek counterparts. For current Internet users, Internet use patterns were significantly different between country groups. When adjusting for gender, age, education and disease duration, country was a significant predictor of frequency of Internet use, eHealth literacy and Interest. The Finnish group of Internet users scored higher in eHealth literacy, while the Greek group of never Internet users had a higher Interest in computer/Internet.

**Conclusions:**

eHealth literacy is either moderate (Finnish group) or low (Greek group). Thus, exposure to ICT and eHealth skills training are needed for this population. Recommendations to improve the eHealth literacy and access to health information among these individuals are provided.

**Electronic supplementary material:**

The online version of this article (10.1186/s12911-017-0531-4) contains supplementary material, which is available to authorized users.

## Background

Schizophrenia spectrum disorders (SSD) are one of the most debilitating forms of mental illness [1], with schizophrenia alone affecting about 1% of the population worldwide [2]. SSD are accompanied by reality distortion, psychotic experiences (e.g. hallucinations and delusions), cognitive impairments, and motivational deficits [3]. These symptoms affect people’s cognition, community functioning [4], daily life [2] and physical health [5–7]. The comprehensive treatment approaches primarily aim to, relieve the wide range of symptoms, enhance persons’ general and psychosocial functioning, and improve their self-management skills and their overall quality of life [8, 9].

Μore than half of people in the spectrum do not receive treatment [10, 11], while those who initially follow treatment, misuse or stop it [2, 12]. This causes a huge personal and economic burden, not only to the person with the illness and his/her family, but also to society [13].

Given the fact that the majority of people with SSD do not get professional help, self-management skills are important [14]. Adequate acquisition of these skills, such as the ability to access, process, and comprehend basic health information and services needed to make appropriate health decisions [15], known as ‘health literacy’ is essential to reading, understanding, and acting on health care information. In this way, good health is maintained and promoted [16]. Understanding relevant health terms and connect health information into the appropriate context, empowers consumers to advance their engagement in self-care activities [17].

The Internet has shown to be a promising and constantly growing source of health information [18, 19]. Between years 2000 and 2016, Internet penetration grew by 900% [20]. Approximately 80% of persons with psychiatric conditions are Internet users [21]. People with SSD use the Internet as every other user does [21–23] to exchange emails, browse Web 2.0 and social media [23], interact with others online [22], seek health information and communicate with peers and health professionals [22–24]. The “ability to seek, find, understand, and appraise health information from electronic sources and apply the knowledge gained to addressing or solving a health problem” is known as ‘eHealth literacy’ [25]. People with SSD search for health information online, although they do not always know which information can be trusted [22]. Therefore, their eHealth literacy needs to be investigated in order to know if their eHealth literacy skills need to be improved, so they will be supported, thus empowered, to find and understand the health information they could read online, in order to improve their self-care activities and health-related decisions.

On the other hand, not all people with SSD are Internet users [23]. It is possible that schizophrenia’s symptoms, such as attention deficit or delusional interpretations [26], and motivational deficits [4] may disrupt Internet use [22]. Not everyone with SSD are willing or able to use the Internet for health-related information due to a lack of computer access, economic problems, difficulties using technology, fear of computer viruses or Internet addiction, a preference for other sources of information, an expectation of low quality of Internet information, their demand for information is satisfied elsewhere, lack of interest, or their wish to rely on a doctor [22]. Thus, investigating this population’s perceptions on important sources of health information, reasons for non-use/discontinuation, and attitudes toward computers/Internet (perceived efficacy and interest on computer/Internet), would provide deeper insight into their preferences and needs related to online – and offline – health information sources [23, 27].

Several studies have examined Internet use patterns, eHealth literacy, and attitudes toward computer/Internet among various, non-mental health-related populations [28–33]. Some studies have explored the Internet use among people with schizophrenia [22, 23] and showed that more than half use the Internet on a daily basis [22], and that the majority of those use it for mental health-related issues [21], while more than one-fourth [23] or less than half [21] use social networking sites. To our knowledge, this is the first study to describe and compare Internet use patterns, eHealth literacy, and attitudes toward computer/Internet among people with SSD from two distant European regions (Finland [FI] and Greece [GR]). The results will provide new insights about this population’s online and offline health information-seeking preferences, and identify their Information and Communication Technology (ICT) training needs, so they could benefit from the positive health-related outcomes of Internet and technology use [34–36]. The following research questions were formed to meet our study goal: Between country groups (Finnish vs. Greek people with SSD): i) What is the prevalence of ICT use (Internet use, mobile phone use, and SMS exchange)?; ii) Among never computer/Internet users, which are their attitudes toward computers/Internet?; iii) Among previous and current computer/Internet users, what is their eHealth literacy level?; iv) Among current computer/Internet users, what is the frequency of Internet use for general, health-related reasons and social networking websites?

The importance of answering the aforementioned questions lies on findings of previous literature, which suggests that people with SSD tend to use ICT in a similar way as the general population [23], and are engaged with social media [23, 37], however their need to access health information is unmet [38]. Nonetheless, their unhealthy lifestyle makes them an appropriate target group for health promotion interventions [39] with the assistance of new technologies [40]. In addition, since health promotion and patient empowerment via ICT have been among the goals of the European agenda [24, 41], it is important to explore these research questions in two EU member states, which are so diverse in their computer/Internet use for general [42] and health-related purposes [43], and mental health resources’ availability and uptake [44, 45]. Exploring the potential differences in ICT-related variables between these two European countries, could contribute to the adaption of the EU eHealth agenda, according to each country’s patients’ needs and level of eHealth literacy. Further, this means that potential differences require individual eHealth actions from each country.

On a country level, this study contributes to the reform of most levels of the mental health care services [46]. From the bottom of the mental health services pyramid [46], self-care, by identifying people’s with SSD health information needs and preferred sources of health information, to the primary health care services, by identifying the prevalence of Internet use for general and health-related purposes, in order to provide online mental health information and support to this population. By acknowledging patients’ Internet use and it’s potential as a (mental) health communication and health promotion tool, the necessity of access to reliable health information and services (e.g. online consultation and psychoeducation) to people with SSD could be recognized.

## Methods

### Design

A cross-sectional survey study was conducted in two countries (Finland and Greece). A descriptive design approach was chosen because a more profound understanding is needed of the experiences and opinions on computer/Internet use (or not use) and Internet use patterns [47] of people with mental illness. These two countries were selected to be compared, as they represent two European extremes on daily Internet use (Finland 85% vs. Greece 55%) [42], and Internet use for health information-seeking (Finland 67% vs. Greece 37%) [43]. As a recent key objective of the European Union is mainstreaming e-mental health among member states [11], country specific characteristics of this population are crucial to be acknowledged, and used as a ground knowledge for future e-mental health plans.

### Setting

One catchment study area (psychiatric clinic) was selected in each country. In Finland, data were collected from outpatient services of two psychiatric clinics in Southern Finland serving 170,000 citizens. In Greece, the data were collected from outpatient services (including outpatient services of the psychiatric clinic’s mobile unit) and inpatient services of a psychiatric clinic of a hospital in Southern Greece, serving a population of approximately 173,450 inhabitants [48].

Both in Finland and Greece, psychiatric treatment for severe and long-term mental health problems - such as SSD – are primarily implemented as outpatient care [49, 50]. People do not stay overnight in the hospital, but they only visit the clinic a few times a week or month to receive treatment (usually a combination of counseling and medication) [51, 52]. Acute inpatient wards provide high-intensity care for seriously ill patients; for example, patients experiencing severe psychotic relapse and behavioral disturbance or patients with high levels of suicidality [53]. The aims of treatment for patients with schizophrenia are to relieve symptoms and to improve patients’ psychosocial functioning and quality of life [54].

### Participants

Study participants were included if they were: 1) 18 years old and above, 2) diagnosed with SSD as a primary diagnosis (F20–29; ICD-10) [55], 3) able to understand, speak and read Finnish (in Finland) or Greek (in Greece), 4) willing to participate in the survey based on their own free will (signed informed consent), 5) considered as stabilized judged by the treating psychiatric nurse (Finland) or psychiatrist (Greece).

### Recruitment

**Fig. 1:**
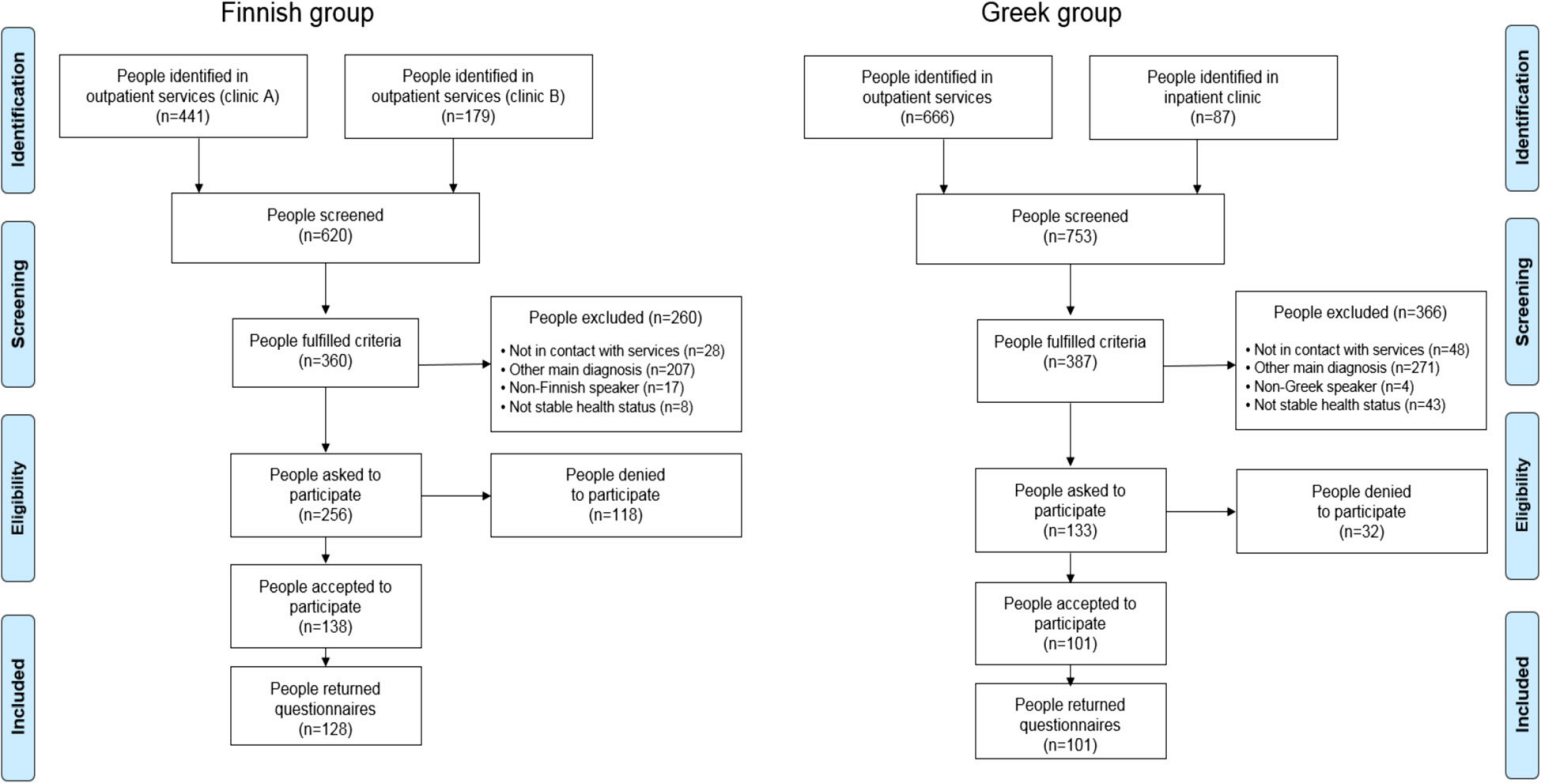
Flow diagram of Finnish and Greek study participants

In Finland potential participants (*N* = 620) who were identified in the records of the outpatient care services of two psychiatric clinics, were screened between 4 June – 16 December, 2015 (Fig. 1). Patient records were reviewed by the psychiatric nurses, who had frequent, scheduled meetings with them during outpatient services. After excluding patients who were not able to participate due to their mental health condition (not stable health status), the rest who met the inclusion criteria, were invited to participate in the study (Fig. 1). The treating nurse gave those who accepted to participate an unsealed envelope containing: an information letter, consent form (2 copies), printed questionnaire, stamps and researcher’s postal address (if participants preferred to return it by post).

In Greece potential participants (*N* = 753) who were identified in the records of the outpatient care services (of the hospital’s clinic or its mobile unit), or admitted for short-term hospitalization to the hospital’s inpatient psychiatric clinic with a stable health status (stabilized and right before release), were screened between 6 September - 5 November, 2015 (Fig. 1). Patient records were reviewed by the treating psychiatrists, and after excluding those patients with not stable health status, the rest who met the inclusion criteria, were eligible to be contacted by the first author in order to be invited to participate in the study. For those who were asked and accepted to participate, the researcher gave them the research material as in the Finnish study.

### Data collection

In Finland all participants filled the questionnaire themselves and returned it to the treating nurse in a sealed envelope, while they had the choice to send it by post to the researcher (*n* = 128, Response rate = 50%). In Greece participants could choose between: filling the questionnaire themselves and returning it to the psychiatric clinic in a sealed envelope (*n* = 3), or filling the questionnaire as a face-to-face structured interview performed by the researcher (*n* = 98) (*n* = 101, Response rate = 76%). The time needed to complete the questionnaire was maximum 30 min. A description of the patient recruitment and the flow of data collection in both country groups is described above in Fig. 1.

### Measures

The data were obtained using a structured questionnaire. The selected questionnaire on ‘Computer and Internet use’ by Choi and DiNitto [28] was chosen as the most appropriate to answer our major research questions, and in addition, it was considered an established, valid and reliable instrument by previous studies [56]. As the questionnaire was used for the first time in a Finnish and Greek native speaking population it was translated, pilot-tested and culturally adapted from the source language (English) to the target languages (Finnish and Greek) based on the ‘Minimal Translation Criteria’ [57]. The translated questionnaires were pilot-tested in 12 Finnish and 5 Greek individuals having the same diagnosis as the target population [58].

All participants answered demographic and basic questions (demographics, computer/Internet use, reasons for non-use/discontinuation, sources of health information) and the respective questionnaire section according to his/her computer/Internet use experience [never-users answered ‘Attitudes Toward Computer/Internet Questionnaire’ (31 items in total), previous users answered ‘eHealth Literacy’ (30 items in total), current users answered ‘eHealth Literacy’ and ‘Internet use patterns’ (36 items in total)]. Details about the questionnaire’s items can be found in the ‘Additional file 1’.

*Sociodemographics* were answered from all study participants and included 7 open or close-ended questions between 2 and 6 alternatives (Additional file 1). *Basic information regarding ICT use and sources of health information* was answered from all study participants and based on Chronaki and colleagues [59] and Choi and DiNitto [28], exploring important sources for health information, ICT use (Internet/Mobile/SMS use), and Reasons for Non-use/Discontinuation.

*Attitudes toward Computer/Internet - ATC/IQ* (Bear and colleagues [60] adapted by Choi and DiNitto [28]) was answered by never computer/Internet users. ATC/IQ measured respondents’ attitudes toward using computers and the Internet, scored on a 5-point Likert scale (‘Completely Disagree’-‘Completely Agree’). It consisted of 10 statements, specifically measuring participants’ efficacy and interest toward computer/Internet (Additional file 1). The internal consistency of the ATC/IQ subscales were found to be good / acceptable. In particular for the Efficacy subscale, Chronbach’s α was .78 in the Finnish sample and α = .80 in the Greek sample. For the Interest subscale, Chronbach’s α was .52 in the Finnish sample and α = .76 in the Greek sample.

*eHealth Literacy Scale* (eHEALS; Norman and Skinner [16] adapted by Choi and DiNitto [28]) was answered by current and previous computer/Internet users. eHEALS measured respondents’ combined knowledge, confidence, and perceived skills in finding, evaluating, and applying electronic health information to health problems [16], scored on a 5-point Likert scale (‘Completely Disagree’-‘Completely Agree’) and consisted of 8 statements (Additional file 1). The internal consistency of the collected data using the eHEALS was good (Cronbach α = .86 in both Finnish and Greek sample), and comparable to reliability estimates reported in previous studies [16, 28]. *Internet use patterns* [28] was answered by current computer/Internet users. It consisted of 6 close-ended questions, between 2 and 12 alternative answers (Additional file 1).

### Data analysis

The data analysis consisted of 8 steps. First, for the items measured by 5-point Likert scales (Important sources of health information, ATC/IQ, eHEALS), the two higher endpoints (“4” and “5” points) were recoded as “Agree” or “Important” or “Useful”, likewise the two lowest endpoints (“1” and “2” points) were recoded as “Disagree” or “Not important” or “Not useful” during the data analysis [24]. In addition, the second item of ATC/IQ (‘Computers/Internet are too complicated for me to understand’), was reverse scored ‘not’ was removed from the original negative statement, after communication with the original author. Second, all data were summarized using descriptive statistics. For normally distributed data mean (standard deviation) was used, while non-normally distributed data were presented as median (minimum, maximum). Categorical data were presented as frequencies and percentages. Third, the final score for efficacy and interest (two major components of ATC/IQ) was the average of all 5 items of each component (score range 1–5), while eHEALS’ final score was the sum of all 8 items (score range 8–40), with the higher scores represented higher levels of efficacy/interest/eHealth literacy. Fourth, comparisons between countries were performed applying 1-way Analysis of Variance (ANOVA) test in case of normally distributed data and Kruskal-Wallis rank test in case of non-normally distributed data. For categorical data, comparisons between countries were performed using Chi-Square test of independence and Fisher’s exact test. Fifth, the level of education was recoded as up to 9 years of formal education, 10–12 years of formal education and >12 years of formal education. The frequency of Internet use was recoded as daily Internet use versus non-daily internet use. Sixth, for binary response variables, multivariable logistic regressions were performed with dependent variables the frequency of Internet use (daily vs. non-daily), the health-related Internet use (yes/no), the social networking websites use (yes/no) and watching videos (yes/no) and independent variable being the country (Greece vs. Finland) adjusting for gender (male, female), age (in years), level of education (up to 9 years of formal education, 10–12 years of formal education and >12 years of formal education) and duration of the disease (in years). Results are reported as Odds Ratios (OR), 95% Confidence Intervals (CI) and *P*-values. Seventh, for continuous response variables, multivariable linear regressions were performed with dependent variables the Efficacy, the Interest and the eHeals and independent variable being the country (Greece vs. Finland) adjusting for the same variables as the logistic regressions above. Results of linear regression models are presented as beta-coefficients (beta), 95% Confidence Intervals (CI) and P-values. Eighth, Internet use for health-related reasons was measured by calculating the prevalence of the three first answer alternatives to the question ‘Reasons of Internet use’ [1) Research health-related information, or 2) Communicate with health professionals about health-related issues, or 3) Communicate with other users about health-related issues) (from ‘Internet use patterns’ section)]. All data was analyzed with the JMP Pro 11, SAS Version 9.4 [61] and SPSS Version 21.0 [62] for Windows. Analyses were considered statistically significant at the *P* < .05 alpha level (2-tailed).

## Results

### Description of participants

In the Finnish group, both genders were almost equally represented, the median age of respondents was 38 years, and median duration of illness was 16 years (Table 1). Most participants were single (*n* = 97, 76%), college or vocational school graduates (*n* = 49, 38%), on disability pension (*n* = 91, 71%), and lived alone (*n* = 87, 68%) (Table 1). In the Greek group, males were the most prevalent, with a median age of 44 years, and a median duration of illness 11 years (Table 1). Most of them were single (*n* = 44, 43%), had completed primary or middle school (*n* = 53, 52%), were unemployed (*n* = 35, 34%), and lived at their parental household (*n* = 43, 42%). When compared to their Finnish counterparts, Greek participants had shorter illness history (*P* = 0.006), the majority of them tended to be older and male (*P* = 0.006), more were in a partnership or married (*P* < .0001), more were unemployed (P < .0001), and much less were living alone (*P* < .0001) (Table 1).

**Table 1:** Sociodemographics and important sources of health information between country groups

Socio-demographic characteristics	Total sample *N* = 229	Finnish group *n* = 128^a^	Greek group *n* = 101^a^	*P* ^*b*^
Age ^+^				
Median (min, max)	41 (19, 77)	38 (20, 64)	44 (19, 77)	0.007
Duration of illness (since the first contact with psychiatric services)				0.006
Median (min, max)^+^	13 (0, 56)	16 (0, 47)	11 (0, 56)	
Gender: N (%)				0.006
Male	132 (58)	61 (48)	71 (70)	
Female	97 (42)	67 (52)	30 (30)	
Marital status: N (%)				<.0001
Single	141 (62)	97 (76)	44 (43)	
Partnership/Married	58 (25)	22 (17)	36 (36)	
Separated/Divorced	26 (11)	9 (7)	17 (17)	
Widowed	4 (2)	0 (0)	4 (4)	
Level of education: N (%)				<.0001
No formal education	3 (1)	3 (2)	0 (0)	
Primary and middle school	73 (32)	20 (16)	53 (52)	
High school	56 (25)	31 (24)	25 (25)	
College or Vocational training	64 (28)	49 (38)	15 (15)	
University degree	33 (14)	25 (20)	8 (8)	
Employment status: N (%)				<.0001
Unemployed	51 (22)	16 (13)	35 (34)	
Social welfare benefit	14 (6)	2 (2)	12 (12)	
Student	10 (5)	7 (5)	3 (3)	
Disability pension	124 (54)	91 (71)	33 (33)	
Employed (including sick leave)	19 (8)	5 (4)	14 (14)	
Other	11 (5)	7 (5)	4 (4)	
Living situation: N (%)				
Own household (with partner/family)	56 (24)	20 (16)	36 (36)	<.0001
Own household (alone)	109 (48)	87 (68)	22 (22)	
Flat share	5 (2)	5 (4)	0 (0)	
Parents’ household	51 (22)	8 (6)	43 (42)	
Supported housing	8 (4)	8 (6)	0 (0)	
Important sources of health information^c^: N (%)				
Internet	121 (53)	76 (59)	45 (45)	0.16
TV/Radio	86 (38)	48 (38)	38 (38)	0.11
Books, medical encyclopaedias and leaflets	117 (51)	61 (48)	56 (55)	0.02
Courses and lectures	100 (44)	41 (32)	59 (58)	<.0001
Newspapers, magazines	76 (33)	48 (38)	28 (28)	0.74
Family, friends, colleagues	141 (62)	72 (57)	69 (68)	0.44
Pharmacies	144 (63)	71 (56)	73 (72)	0.04
Face-to-face contact with medical professionals	203 (89)	111 (87)	95 (94)	0.85

^a^Percentages inside the parentheses (%) refer to percentages within country

^b^Fisher’s Exact Test

^c^Number of the highest points (‘4 = Important’ and ‘5 = Very important’) coded together

^+^Kruskal-Wallis rank test used for comparisons between countries

Among the Finnish group, the most important source of health information was face-to-face contact with medical professionals, followed by the Internet, family, friends and colleagues (Table 1). Among the Greek group, the most important source of health information was face-to-face contact with medical professionals, followed by pharmacies; and family, friends and colleagues (Table 1). As opposite to Finnish participants, more patients in Greece favored courses and lectures (*P* < .0001), books, medical encyclopaedias and leaflets (*P* = 0.02); and pharmacies (*P* = 0.04) as a health information source (Table 1).

### ICT use, ATC/IQ, and eHealth literacy

As for ICT use, most Finnish participants (*n* = 111, 87%) were current Internet users, 2% (*n* = 3) were previous Internet users, and 11% (*n* = 14) had never used the Internet (Table 2). On the contrary, over a half of the Greek participants (*n* = 60, 59%) had never used the Internet, 8% (*N* = 8) were previous Internet users and a third (*n* = 33, 33%) were current Internet users (*P* < .0001) (Table 2). The reported reasons for never use/discontinuation were different between country groups (*P* < .0001): No Internet connection and/or computer at home because of cost (FI = 9 vs. GR = 14); It is not helpful (FI = 0, GR = 1); I do not need it (FI = 6 vs. GR = 19); Cannot use computer because of disability/pain (FI = 0 vs. GR = 18); Other reason (FI = 2 vs. GR = 16). In the Finnish group, other reasons included: 1) lack of knowledge in using computers/Internet and 2) broken Internet connection/computer. In the Greek group, other reasons included: 1) afraid of breaking it; 2) no time to engage with it; 3) very difficult to use; 4) I don’t know how to use it; 5) prefer to spend my free time with friends and family; 6) relative uses it for me; 7) unaware of what Internet is and 8) it is confusing.

Attitudes Toward Computer/Internet (ATC/IQ) among never Internet users were found to be neutrally scored among Finnish participants (Table 2). The first component of ATC/IQ, efficacy, had a mean score of 2.93 out of maximum 5. The second component of ATC/IQ, interest, had a mean score of 2.60 out of maximum 5. As for the Greek participants, efficacy was slightly higher compared to their Finnish counterparts (Mean = 3.06; *P* = 0.585). Further, interest scored significantly higher in the Greek group (Mean = 3.16; *P* < .0001) (Table 2). The supplementary items of this section, denoted similar willingness to find online health information (*P* = 0.829) and comfort joining online health discussion groups between countries (*P* = 0.207) (Table 2).

**Table 2:** ICT use, ATC/IQ and eHealth literacy between country groups

	Finnish group	Greek group	*P-value*
	n (%)	n (%)	
Information and Communication Technology use (among all participants):			
Internet use			<.0001
Never user	14 (11)	60 (59)	
Previous user	3 (2)	8 (8)	
Current user	111 (87)	33 (33)	
Mobile phone use			0.01
Yes	123 (96)	85 (84)	
No	5 (4)	16 (16)	
SMS use			<.0001
Yes	109 (85)	44 (44)	
No	19 (15)	57 (56)	
Attitudes Towards Computers/Internet (among never Internet users)^*^:	Mean (SD)	Mean (SD)	*P*-value
ATC/IQ Efficacy	2.93 (0.81)	3.06 (0.86)	0.585
ATC/IQ Interest	2.60 (0.67)	3.16 (0.50)	<.0001
Supplementary questions^+^:	Median (Min, max)	Median (Min, max)	*P*-value
Willingness to find online health information if someone teaches me how to use the Internet	4 (1, 5)	4 (1, 4)	0.829
Comfort joining an online health discussion group and exchange emails with others	2.5 (1, 5)	3 (1, 5)	0.207
	Mean (SD)	Mean (SD)	*P*-value
eHealth literacy (among previous and current Internet users)	27.05 (5.36)	23.15 (7.23)	<.0001
Supplementary questions:	Median (Min, max)	Median (Min, max)	*P*-value
Perceived usefulness	4 (1, 5)	3 (1, 5)	0.199
Perceived importance	4 (1, 5)	4 (1, 5)	0.732
Comfort joining an online health discussion group and exchange emails with others	2 (1, 5)	3 (1, 5)	0.492

^*^One-way ANOVA test was used to detect differences between countries

^+^Kruskal-Wallkis rank test was used to detect differences between countries

The mean score of the eHealth literacy (eHEALS) was significantly higher amongst Finnish Internet users compared to Greek Internet users [27.05 (SD 5.36) vs. 23.15 (SD 7.23); *P* < .0001; Table 2]. The supplementary items of this section were compared between country groups, and no statistically significant differences were found (Table 2).

### Internet use patterns and activities

Almost all Finnish Internet users (*n* = 106, 95%) had a home Internet access and email address (Table 3). About two-thirds were using the Internet at least once a day. The most common reasons for Internet use were: online banking/pay bills (*n* = 101, 91%), research for information about topics of interest (*n* = 98, 88%) (not health-related), and send/receive email (*n* = 91, 82%). A bit less than two-thirds (*n* = 67, 60%) believed that it is sometimes easy for them to find the website they wanted and trace the information they needed from a specific site. The most common reason for making Internet use harder for them was difficulty in concentration for long periods of time (*n* = 31, 28%) (Table 3).

**Table 3:** Internet patterns among Finnish and Greek current Internet users

Internet patterns	Internet users			
N (%)	Total *N* = 144 Ν (%)	Finnish group *n* = 111 ^a^ n (%)	Greek group *n* = 33 ^a^ n (%)	*P* - value
Location of Internet access:^b^				
Home	133 (92)	106 (95)	27 (82)	0.02
Apartment complex	1 (1)	1 (1)	0 (0)	–
Family/friend’s house	3 (2)	3 (3)	0 (0)	–
Library	1 (1)	1 (1)	0 (0)	–
Other	6 (4)	0 (0)	6 (18)	<.0001
Email address:				0.28
Yes	133 (92)	104 (94)	29 (88)	
No	11 (8)	7 (6)	4 (12)	
Frequency of Internet use:				0.03
At least once a day	108 (75)	85 (77)	23 (70)	
Every few days	15 (10)	9 (8)	6 (18)	
Once a week	8 (6)	7 (6)	1 (3)	
A few times a month	11 (8)	9 (8)	2 (6)	
Once a month or less	2 (1)	1 (1)	1 (3)	
Reasons of Internet use:				
Research health-related information	81 (56)	61 (55)	20 (61)	0.57
Communicate with health professionals about health-related issues	9 (6)	5 (4)	4 (12)	0.21
Communicate with other users about health-related issues	15 (10)	10 (9)	5 (15)	0.34
Internet use for health-related purposes^c^	81 (56)	61 (55)	20 (61)	0.56
Research information about other topics or issues of interest to me	128 (89)	98 (88)	30 (91)	0.67
Send/receive email	109 (76)	91 (82)	18 (55)	0.001
Buy products online	84 (58)	69 (62)	15 (45)	0.09
Do banking online/pay bills	110 (76)	101 (91)	9 (27)	<.0001
Read news, papers, magazines, and books online	102 (71)	79 (71)	23 (70)	0.87
Play games online	45 (31)	26 (23)	19 (58)	<.0001
Watch videos (including YouTube)	111 (77)	81 (73)	30 (91)	0.03
Use social networking websites and/or dating sites	86 (60)	61 (55)	25 (76)	0.03
Other	28 (19)	17 (15)	11 (33)	0.02
Easiness to locate website and to find information within that site:^d^				0.02
Always easy	42 (29)	30 (27)	12 (37)	
Sometimes easy	75 (52)	67 (60)	8 (24)	
Not so easy	22 (16)	13 (12)	9 (27)	
Difficult	2 (1)	1 (1)	1 (3)	
Very difficult	3 (2)	0 (0)	3 (9)	
Problems hardening Internet use:				
Pain in limbs	4 (3)	3 (3)	1 (3)	0.93
Unsteady hands	13 (9)	9 (8)	4 (12)	0.49
Difficulty concentrating for long periods of time	42 (29)	31 (28)	11 (33)	0.57
Difficulty sitting for long periods of time	29 (20)	19 (17)	10 (30)	0.10
Eyes that tire easily	27 (19)	17 (15)	10 (30)	0.06
Other problems	27 (18)	21 (19)	6 (18)	0.91

^a^Percentages inside the parentheses (%) refer to percentages within country

^b^Fisher’s Exact Test

^c^Prevalence of the three first answer alternatives to the question ‘Reasons of Internet use’

^d^ *P*-value: Pearson’s Chi Square

On the other hand, although the majority of the Greek group of Internet users had a home Internet access (*n* = 27, 82%), close to about a fifth (n = 6, 18%) had Internet access through other places (Table 3). Most of them (*n* = 29, 88%) had an email address, were using the Internet at least once per day (*n* = 23, 70%), and stated it always easy to find the website and the information they were looking for on the Internet (*n* = 12, 37%) (Table 3). The most common reasons for Internet use among Greek participants included: research for information about topics of interest (not health-related) (*n* = 30, 91%), watching videos (n = 30, 91%), and use of social networking websites/dating sites (*n* = 25, 76%). The most frequent reason that made Internet use harder for them, was difficulty in concentration for long periods of time (*n* = 11, 33%) (Table 3).

When comparing the Finnish with the Greek group, statistically significant differences were found for: location of Internet access, the frequency, the reasons of Internet use, easiness to locate information, and various reasons for Internet use (Table 3).

In the Finnish group, more than the half participants used the Internet for health-related purposes (*n* = 61, 55%) and the same percentage for social networking and dating websites (n = 61, 55%), while many of them used if to watch videos (*n* = 81, 73%). Similarly, in the Greek group 61% (*n* = 20) used the Internet for health-related reasons, many used it for social networking and dating sites (n = 25, 76%) and the majority for watching videos (n = 30, 91%). Statistically significant differences between groups were identified for: Internet use for social networking and dating sites (*P* = 0.03), watching videos (*P* = 0.03), play games online (*P* < .0001), send/receive email (*P* = 0.001), and do banking online/pay bills (*P* < .0001) (Table 3).

Last, since the sample was not matched for disease duration, we included disease duration as a confounder in all the regression models. This information can be found in the supplementary tables in Additional file 2.

### Internet use patterns, eHealth literacy, and attitudes toward computer/ internet, multivariable analysis

Table 4 summarizes the impact of country as an independent variable in several linear and logistic multivariable regression models. All models were adjusted for the same demographic confounding variables (gender, age, level of education and duration of the disease). Results indicated that Finnish participants had much higher odds of being daily Internet users compared to Greek patients (OR 14.52; 95% Confidence Interval [CI] from 5.40 to 39.03; *P* < .0001). Country was also a significant predictor of Interest (beta = −0.44; 95% CI from −0.75 to −0.13; *P* = 0.006) amongst never Internet users, and eHealth literacy (beta = 5.02; 95% CI from 2.52 to 7.53; *P* < .0001) among Internet users, No significant associations were identified between internet use for health-related purposes, social networking/dating websites, watching videos, Efficacy, and country.

**Table 4:** A summary of odds ratios or beta coefficients reporting the association between country and several dependent variables of interest assessed bymultiple regression models

Dependent Variable	Country ^c^ Odds Ratio^a^ / Beta^b^	95% CI	*P*-value
Among current Internet users:			
Frequency of Internet use (daily vs. non-daily)^a^	14.52	5.40 to 39.03	<.0001
Internet use for:			
Health-related purposes (yes/no)^a^	0.87	0.35 to 2.20	0.776
Social networking and dating websites (yes/no)^a^	0.45	0.15 to 1.33	0.150
Watching videos (yes/no)^a^	0.53	0.13 to 2.17	0.380
Among previous & current Internet users:			
eHealth literacy^b^	5.02	2.52 to 7.53	<.0001
Among never Internet users:			
Efficacy^b^ (ATC/IQ component)	−0.06	−0.66 to 0.44	0.800
Interest^b^ (ATC/IQ component)	−0.44	−0.75 to −0.13	0.006

^a^Logistic regression

^b^Linear regression

^c^Adjusted for gender, age, level of education and duration of the disease. Greece was used as a reference category

## Discussion

### Key findings

This study was the first to describe and compare Internet use for general and health-related purposes, eHealth literacy, and attitudes toward computer/Internet among Finnish and Greek adults with SSD. The majority of Finnish participants were computer/Internet users, tended to use the Internet more frequently, and scored higher in eHealth literacy, when compared to their Greek counterparts. Most of Greek participants were never Internet users, while never-users exhibited higher interest in computers/Internet when compared to their Finnish counterparts. Internet users from both groups showed similar use patterns concerning: Internet use for health-related purposes, social media/dating websites use and watching videos. More than half of Internet users, used the Internet for health/related purposes.

### Discussion under the light of the literature

As expected, Internet use was much more prevalent in the Finnish than in the Greek group, which is in accordance with the general population [42]. The biggest percentage of the Greek participants had never used the Internet. However, surprisingly enough, considering the economic situation in Greece [63], cost was not the first reason for non-usage for the Greek population (as for Finns), but their perception of not needing the Internet. This could be explained due to the big differences between the two country samples in relation to education and income [64], culture [65], weather and latitude affecting significantly the duration of the day and lifestyle parameters including, spending more time out of the house, having more social contacts and social interactions [66–68], which surely affect Internet use from home.

Among Internet users, Finns scored significantly higher in eHealth literacy. This could mean that Greeks were unsure about their competence regarding skills required to effectively engage information technology for health, which could further mean they were, indeed, not competent enough. Thus, not only the majority of Finns used the Internet, but also those who used the Internet, appeared more confident regarding their health-related ICT skills. Level of education, culture, language, and ethnicity are factors affecting eHealth literacy, which could explain the significant different scores between country groups [17, 25, 69]. Since the eHealth literacy score of the study participants was moderate or low, it means that they did not have the appropriate skills to find, understand and appraise the health information they read online, thus they could not use this source of health information to deeper understand their illness and use it to develop their self-management skills and further improve their health.

While the use of the Internet for health information has been somewhat less common in the south European countries [70], in our study the south European (Greek group) and north European participants (Finnish group) reported similar use of Internet for health-related purposes. Nevertheless, there was considerable variation in the importance placed on the Internet as a source of health information. In the Finnish group, the Internet was considered the second most important source, preceded by health professionals. In contrast, the Greek group considered the Internet to be the least important source of health information. This finding is in line with a previous European study [24] where health professionals were the most important source of health information, followed by family, friends and colleagues, while Internet was the second least important source of health information. In a more recent European study [41], the lowest agreement rate to the statement ‘I know how to use the health information I find on the Internet to help me’ (eHEALS item) was, again, found in the Greek population (84%), where the highest agreement rate was reported by Finns (96%) [41], supporting our current findings (39% vs. 51%).

Greek never-Internet users, exhibited considerably higher Interest towards computers/Internet than their Finnish counterparts. Based on the items of this component, the Greek group appears more willing to learn, read and hear about computers/Internet. This might be explained by the limited exposure to ICT among the Greek population comparing to the Finnish [71]. Which could further mean that more Finns are aware of computers and the Internet and they are consciously choosing not to use them, whereas Greeks would like to learn more about computers and the Internet if they had the chance.

More than half of the participants in both country groups, used the Internet for health-related purposes. A German study among psychiatric patients, reported even higher prevalence of Internet use for mental health-related reasons [21]. Notwithstanding, the Internet users of the Greek group appeared to use social networking sites and YouTube more than their Finnish counterparts. A recent study among the general population [72] reports slightly different results, with 33% of Greeks and 47% of Finns using social network websites, and 14% of Greeks and 19% of Finns using multimedia content sharing websites (including YouTube). However, a latter finding shows that among the general population, social media use may be highest among those with lower income [37], which might explain our results.

### Strengths and limitations

A possible limitation of this study is that the subjects from each country were from one catchment area of a specific clinic in a selected geographic location with a limited number of subjects, and therefore, generalizability may be limited. Additionally, the Greek participants were recruited from both urban (clinic’s outpatient services) and rural areas (clinic’s mobile unit) where exposure to ICT is not the same as in urban areas which could induce bias. Second, the selection of the participants was not random, thereby the sample is not representative of the population and might suffer from selection bias. Third, although the study protocol was the same in both countries, and the selected instrument was validated and reliable certain adaptions needed to be made according to each clinic’s operation procedures. For example, in Finland psychiatric nurses were responsible for patient recruitment, whereas in Greece psychiatrists were responsible for recruitment. Thus, a recruitment bias might have been introduced. A fourth limitation is that patients’ symptom severity and psychosocial functioning were not assessed, thus we could not assess if severity and functioning affected eHealth literacy, attitudes and use of computers and Internet. However, psychiatric nurses (Finland) and psychiatrists (Greece) referred only stable patients for potential participation to the study. Fifth, this population exhibits motivational deficits [4], which could affect their motives in using computers/Internet for general and health-related reasons. Thus, this parameter needs further investigation. Sixth, participants’ eHealth skills (eHealth literacy) were evaluated based on their subjective self-report, without these skills being assessed, tested or actually observed, thus not objectively measured [17]. Seventh, almost all Greek participants preferred to complete the questionnaire as an interview, thus, the answers could suffer from interviewers’ bias. Similarly, social desirability bias cannot be excluded. However, to minimize other interview biases, CA followed appropriate interview guidelines [73]. Eighth, sociodemographic characteristics such as family structure were not assessed, although it could be a factor affecting computers/Internet use, for example having a family member looking for health information online instead of the person with SSD. For that reason multivariable analyses adjusted by socio-demographics such as gender, age, level of education and duration of the disease were performed. Ninth, although robustness tests were performed in our multivariable regressions, their results should be interpreted with caution since they involve limited sample sizes. Despite the aforementioned limitations, this study provides several strengths. Many Internet use aspects were examined providing new information about the experiences, opinions and attitudes toward computers/Internet of those with SSD. In addition, for the first time eHealth literacy of people with SSD from two country groups was described and compared. This provided valuable insights of eHealth literacy of two groups of people with SSD who live in two distant European regions, which represent two extremes in Internet use and experience.

### Impact of the study

Our study could raise awareness about the low/moderate eHealth literacy performance of our population. As suggested by the World Health Organization [74] low eHealth literacy can negatively affect people and society, causing poor health outcomes, increasing rates of chronic disease, health care costs and health information demands, and prevents equity. Health literacy is more than being able to read pamphlets and successfully make appointments with health professionals. By improving people’s access to health information and their capacity to use it effectively, health literacy is critical to empowerment [16]. Empowering people with SSD could lead to personal autonomy in health-related decision making [75]; responsibility of their health care which controls health costs [75, 76]; and improvement of their health outcomes [75, 77]. Knowing these people’s ICT experiences and preferences could contribute to the reform of health care systems, increasing access to health information, services, support and health education via new technologies, while doing so in accordance with patients’ needs. More specifically, based on our results, treatment and self-management skills of people wih SSD could be improved by, enhancing their eHealth literacy, thus, enabling them to find, understand and apply to their own health, the health information which are available online. Additionally, by creating high quality (mental) health information and uploading it online in popular websites and social media.

## Conclusions

The goal of the study was to investigate eHealth use among adults with SSD in two distant European countries, Finland and Greece, and to compare the country groups. Governmental agendas aim to enhance citizens’ eHealth literacy. Shedding light in people’s with SSD Internet use patterns and eHealth literacy, explores the potential of ICT use for health-related purposes of this marginalized group. The main findings of our study showed that the majority of Finns with SSD were Internet users, while more than half, used the Internet for health-related purposes. Their eHealth literacy was found to be moderate. On the other hand, a third of Greeks with SSD used the Internet, while the majority used the Internet for health-related purposes. Their eHealth literacy was found to be significantly lower than their Finnish counterparts. Low Internet use among the Greek group and its significant difference to the Finnish group, suggests that different approaches for the improvement of health literacy should be adopted in different EU states. Since for the Greek group the most important health information sources were health professionals and pharmacies, Greek patients could be reached through these means in order to distribute online health resources/programs and mental health literacy trainings. In the case of the Finnish group, this could be applied through health professionals and the Internet. Clearly, eHealth literacy of people with SSD needs to be improved in both countries. By being eHealth literate, people with SSD could consciously be informed about the health issues of their interest, decide which options fit them best and manage their health. People with SSD who never used the Internet reported interest toward computer/Internet, with significantly higher interest among the Greek group. Thus, exposure to computers and Internet by installing the appropriate equipment in areas of easy access to this population and eHealth literacy trainings, could offer to people with SSD an understanding of how technology can be used effectively, in order to have a positive impact on their health management. Finally, since the plethora of Internet users in both countries used social networking sites, this could potentially be another mean of communicating (mental) health information.

Future studies could test which devices are preferable for people with SSD and measure the impact in patients’ health management after offering the appropriate equipment and eHealth literacy training. The aspect of eHealth literacy should be further investigated as it is influenced by many factors, including motivation for information-seeking. Finally, studies could investigate the distribution of mental health information and training about (mental) health management and promotion via social networking sites and videos.

## Additional files

Additional file 1: Questionnaire’s items. (DOCX 24 kb)

Additional file 2: Tables with the full regression models. (DOCX 20 kb)

## Abbreviations

ATC/IQ: Attitudes Toward Computer/Internet Questionnaire
eHEALS: eHealth Literacy Scale
EU: European Union
FI: Finland
GR: Greece
ICT: Information and Communication Technology
SMS: Short Message Service
SSD: Schizophrenia Spectrum Disorders
