# Supplementary material for: Internet use, eHealth literacy and attitudes toward computer/internet among people with schizophrenia spectrum disorders: a cross-sectional study in two distant European regions
Source: BMC Med Inform Decis Mak. 2017 Sep 20;17:136. doi: 10.1186/s12911-017-0531-4 (PMC5607489; doi:10.1186/s12911-017-0531-4)
Supplement: Supplementary file 1 — Questionnaire’s items. (DOCX 24 kb) [file 12911_2017_531_MOESM1_ESM.docx]

## Additional File 1: Questionnaire’s items

*Sociodemographics*:

1) Age (open-ended question);

2) Gender (‘Male’, ‘Female’);

3) Marital status (‘Single’, ‘Partnership/Married’, ‘Seperated/Divorced’, ‘Widowed’);

4) Level of education (‘No formal education’, ‘Primary and middle school’, ‘High school’, ‘College or Vocational training’, ‘Bachelor’s degree’, ‘Master’s or Doctoral degree’);

5) Employment status [‘Unemployed’, ‘Social welfare benefit’, ‘Student’, ‘Disability pension’, ‘Employed (including sick leave)’, ‘Other’];

6) Household occupancy [‘Own household (with partner/family)’, ‘Own household (alone)’, ‘Flat share’, ‘Parents’ household’, ‘Supported housing’];

7) Age of first contact with psychiatric services (open question).

*Basic information regarding ICT use and sources of health information:*

1) *Internet use* [3 close-ended alternatives (‘Never user’, ‘Previous user’, ‘Current user’)] and *Reasons for non-use / discontinuation* [5 alternatives, 4 close-ended and 1 open-ended (‘No Internet connection and/or computer at home because of cost’, ‘It is not helpful’, ‘I do not need it’, ‘Cannot use computer because of disability or pain’, ‘Other reasons’)], 3) *Mobile phone use* and *SMS use* provided dichotomous alternatives (‘Yes’, ‘No’).

*2*) *Important sources of health information* [57] composed of 8 items measuring the perceived importance of 8 sources of health information, scored on a 5-point Likert scale (‘Not Important’-‘Important’) with an additional option ‘6’ indicated ‘Do not know’ (Internet, TV/radio, Books/medical encyclopaedias/leaflets, Courses/Seminars, Newspapers/Magazines, Family/Friends/ Colleagues, Pharmacies, Face-to-face contact with medical professionals);

*Attitudes toward Computer/Internet - ATC/IQ*:

Efficacy:

1) ‘I know that if I worked hard to learn about computers/Internet, I could do well’ (‘Completely Disagree’-‘Completely Agree’);

2) ‘Computers/Internet are too complicated for me to understand’ (‘Completely Disagree’-‘Completely Agree’);

3) ‘I think I am the kind of person who would learn to use a computer/Internet well’ (‘Completely Disagree’-‘Completely Agree’);

4) ‘I think I am capable of learning to use a computer/Internet’ (‘Completely Disagree’-‘Completely Agree’);

5) ‘Given a little time and training, I know I could learn to use a computer/Internet’) (‘Completely Disagree’-‘Completely Agree’)

Interest:

1) ‘Learning about computers/Internet is a worthwhile and necessary subject’;

2) ‘Reading or hearing about computers/ Internet would be (is) boring’;

3) ‘I don’t care to know more about computers;

4) Computers/Internet would be (are) fun to use’;

5) Learning about computers/ Internet is a waste of time’) toward computers/Internet.

In addition, in this part of the questionnaire participants answered 2 additional items [26]:

1) Willingness to use online health information; and

2) Comfort with joining online health discussion groups and exchanging emails with other participants; both scored on a 5-point Likert scale (‘Completely Disagree’-‘Completely Agree’), where higher scores suggest greater levels of willingness/comfort.

*eHealth Literacy Scale*:

1) ‘I know what health resources are available on the Internet’ (‘Completely Disagree’-‘Completely Agree’);

2) I know where to find helpful health resources on the Internet’ (‘Completely Disagree’-‘Completely Agree’);

3) ‘I know how to find helpful health resources on the Internet’ (‘Completely Disagree’-‘Completely Agree’);

4) ‘I know how to use the Internet to answer my questions about health’ (‘Completely Disagree’-‘Completely Agree’);

5) ‘I know how to use the health information I find on the Internet to help me’ (‘Completely Disagree’-‘Completely Agree’);

6) ‘I have the skills I need to evaluate the health resources I find on the Internet’ (‘Completely Disagree’-‘Completely Agree’);

7) ‘I can tell high quality health resources from low quality health resources on the Internet’ (‘Completely Disagree’-‘Completely Agree’);

8) ‘I feel confident in using information from the Internet to make health decisions’ (‘Completely Disagree’-‘Completely Agree’).

In addition, in this part the questionnaire participants also answered 3 more questions indicating their opinion about:

1) ‘I would be comfortable joining an online health discussion group and exchange emails with other participants’ (‘Completely Disagree’-‘Completely Agree’);

2) Usefulness of using the Internet for health-related decisions (‘Not at all Useful’-‘Very Useful’); and

3) Importance of access to sources of health information (‘Not at all Important’-‘Important’) a with higher scores suggesting higher levels of confidence / comfort.

*Internet use patterns*:

1) Location of Internet access (Home, Apartment complex, Family/friend’s house, Library, Other),

2) Existence of email address (‘Yes’, ‘No’),

3) Frequency of Internet use (‘At least once a day’, ‘Every few days’, ‘Once a week’, ‘A few times a month’, ‘Once a month or less than that’),

4) Reasons of Internet use [‘Research health-related information’, ‘Communicate with health professionals about health-related issues’, ‘Communicate with other users about health-related issues’, ‘Research information about other topics or issues of interest to me’, ‘Send/receive email’, ‘Buy products online’, ‘Do banking online/pay bills’, ‘Read news, papers, magazines, and books online’, ‘Play games online’, ‘Watch videos (including YouTube)’, ‘Use social networking websites and/or dating sites (e.g. Facebook.com, soumi24.fi/zoo.gr)’, ‘Other’],

5) Difficulty in finding a website and searching for information (‘Always easy’, ‘Sometimes easy’, ‘Not so easy’, ‘Difficult’, ‘Very difficult’),

6) Physical restrictions which make Internet use difficult (‘Pain in limbs’, ‘Unsteady hands’, ‘Difficulty concentrating for long periods of time’, ‘Difficulty sitting for long periods of time’, ‘Eyes that tire easily’, ‘Other problems’).
