# Supplementary material for: Internet use, eHealth literacy and attitudes toward computer/internet among people with schizophrenia spectrum disorders: a cross-sectional study in two distant European regions
Source: BMC Med Inform Decis Mak. 2017 Sep 20;17:136. doi: 10.1186/s12911-017-0531-4 (PMC5607489; doi:10.1186/s12911-017-0531-4)
Supplement: Supplementary file 2 — Tables with the full regression models. (DOCX 20 kb) [file 12911_2017_531_MOESM2_ESM.docx]

| Table 1. Logistic regression predicting the odds of frequency of internet use (daily versus non-daily) among users with independent variables gender, age, level of education, duration of the disease and country of observation | | | |
| --- | --- | --- | --- |
| **Variable** | **Odds Ratio** | **95% CI** | **p-value** |
| **Female gender** | 1.113 | 0.459 to 2.70 | 0.812 |
| **Age** | 0.849 | 0.803 to 0.899 | <0.0001 |
| **Level of education** | 1.864 | 1.274 to 2.728 | 0.001 |
| **Duration of disease** | 1.019 | 0.972 to 1.066 | 0.431 |
| **Finland (country)** | 14.52 | 5.404 to 39.03 | <0.0001 |
| Chi-square 130.72 on d.f. 5, p-value<0.0001, pseudo-R^2^ = 0.476. n=203 included in the analysis | | | |

| Table 2. Linear regression with dependent variable the efficacy among non-internet users and independent variables gender, age, level of education, duration of the disease and country of observation | | | |
| --- | --- | --- | --- |
| **Variable** | **Beta** | **95% CI** | **p-value** |
| **Female gender** | -0.057 | -0.475 to 0.361 | 0.786 |
| **Age** | -0.025 | -0.044 to -0.005 | 0.013 |
| **Level of education** | 0.152 | -0.052 to 0.357 | 0.141 |
| **Duration of disease** | 0.001 | -0.016 to 0.017 | 0.994 |
| **Finland (country)** | -0.064 | -0.656 to 0.437 | 0.800 |
| F=1.746, p-value=0.136, R^2^=0.114 | | | |

| Table 3. Linear regression with dependent variable the interest among non-internet users and independent variables gender, age, level of education, duration of the disease and country of observation | | | |
| --- | --- | --- | --- |
| **Variable** | **Beta** | **95% CI** | **p-value** |
| **Female gender** | 0.044 | -0.215 to 0.303 | 0.737 |
| **Age** | 0.001 | -0.012 to 0.013 | 0.951 |
| **Level of education** | -0.022 | -0.148 to 0.105 | 0.733 |
| **Duration of disease** | 0.001 | -0.010 to 0.011 | 0.916 |
| **Finland (country)** | -0.442 | -0.752 to -0.131 | 0.006 |
| F=1.746, p-value=0.136, R^2^=0.114 | | | |

| Table 4. Linear regression with dependent variable the eHeals among internet users and independent variables gender, age, level of education, duration of the disease and country of observation | | | |
| --- | --- | --- | --- |
| **Variable** | **Beta** | **95% CI** | **p-value** |
| **Female gender** | -0.414 | -2.555 to 1.726 | 0.702 |
| **Age** | -0.013 | -0.155 to 0.130 | 0.861 |
| **Level of education** | -0.764 | -1.687 to 0.158 | 0.104 |
| **Duration of disease** | -0.079 | -0.229 to 0.070 | 0.295 |
| **Finland (country)** | 5.024 | 2.519 to 7.528 | <0.0001 |
| F=3.583, p-value=0.105, R^2^=0.124 | | | |

| Table 5. Logistic regression predicting the odds of health-related internet use (yes/no) among users with independent variables gender, age, level of education, duration of the disease and country of observation | | | |
| --- | --- | --- | --- |
| **Variable** | **Odds Ratio** | **95% CI** | **p-value** |
| **Female gender** | 1.710 | 0.797 to 3.669 | 0.168 |
| **Age** | 0.970 | 0.930 to 1.011 | 0.154 |
| **Level of education** | 1.145 | 0.822 to 1.595 | 0.424 |
| **Duration of disease** | 1.034 | 0.978 to 1.093 | 0.243 |
| **Finland (country)** | 0.875 | 0.349 to 2.197 | 0.776 |
| Chi-square 4.791 on d.f. 5, p-value=0.442, pseudo-R^2^ = 0.036. n=123 included in the analysis | | | |

| Table 6. Logistic regression predicting the odds of social networking internet use (yes/no) among users with independent variables gender, age, level of education, duration of the disease and country of observation | | | |
| --- | --- | --- | --- |
| **Variable** | **Odds Ratio** | **95% CI** | **p-value** |
| **Female gender** | 1.193 | 0.517 to 2.755 | 0.680 |
| **Age** | -0.084 | 0.845 to 0.959 | 0.001 |
| **Level of education** | 1.004 | 0.704 to 1.432 | 0.982 |
| **Duration of disease** | 1.021 | 0.962 to 1.085 | 0.494 |
| **Finland (country)** | 0.452 | 0.153 to 1.332 | 0.150 |
| Chi-square 22.837 on d.f. 5, p-value<0.001, pseudo-R^2^ = 0.169. n=123 included in the analysis | | | |

| Table 7. Logistic regression predicting the odds of watching videos as internet use (yes/no) among users with independent variables gender, age, level of education, duration of the disease and country of observation | | | |
| --- | --- | --- | --- |
| **Variable** | **Odds Ratio** | **95% CI** | **p-value** |
| **Female gender** | 0.320 | 0.115 to 0.890 | 0.029 |
| **Age** | 0.938 | 0.880 to 0.998 | 0.048 |
| **Level of education** | 0.913 | 0.611 to 1.362 | 0.654 |
| **Duration of disease** | 0.977 | 0.919 to 1.039 | 0.463 |
| **Finland (country)** | 0.534 | 0.132 to 2.168 | 0.380 |
| Chi-square 22.837 on d.f. 5, p-value<0.001, pseudo-R^2^ = 0.176. n=123 included in the analysis | | | |

| Table 8. Summary table containing the Odds Ratios or beta coefficients of various multiple regressions (logistic/linear) with dependent variables presented 1^st^ column and independent variable the country (Greece used as reference category; second column) adjusting for gender, age, level of education and duration of the disease. | | | |
| --- | --- | --- | --- |
| **Dependent Variable** | **Country**  **Odds Ratio* / Beta^†^** | **95% CI** | **p-value** |
| **Frequency of internet use (daily versus non-daily)^*,1^** | 14.52 | 5.404 to 39.03 | <0.0001 |
| **Efficacy^†,2^** | -0.064 | -0.656 to 0.437 | 0.800 |
| **Interest^†,3^** | -0.442 | -0.752 to -0.131 | 0.006 |
| **eHeals^†,4^** | 5.024 | 2.519 to 7.528 | <0.0001 |
| **Health-related internet use (yes/no)^*,5^** | 0.875 | 0.349 to 2.197 | 0.776 |
| **Social networking internet use (yes/no)^*,6^** | 0.452 | 0.153 to 1.332 | 0.150 |
| **watching videos as internet use (yes/no)^*,7^** | 0.534 | 0.132 to 2.168 | 0.380 |
| Models ^1,4,5,6,7^ are among internet users  Models ^2,3^ are among non-internet users | | | |
